# Development of a dehydrated fortified food base from fermented milk and parboiled wheat, and comparison of its composition and reconstitution behavior with those of commercial dried dairy‐cereal blends

**DOI:** 10.1002/fsn3.1226

**Published:** 2019-10-15

**Authors:** Ashwini V. Shevade, Yvonne C. O'Callaghan, Nora M. O'Brien, Thomas P. O'Connor, Timothy P. Guinee

**Affiliations:** ^1^ Teagasc Food Research Centre Moorepark Fermoy Ireland; ^2^ School of Food and Nutritional Sciences University College Cork Cork Ireland

**Keywords:** composition, consistency, dairy‐cereal blends, pasting viscosity, rheology

## Abstract

Dehydrated blends of milk and cereal are reconstituted and consumed as a nutritious soup or porridge in many regions; the composition and reconstitution behavior of the blends are likely to impact on nutritional quality and consumer acceptability of the soup/porridge. Experimental samples of dried fermented milk‐bulgur wheat blend (FMBW) and commercial samples of dried dairy‐cereal blends, namely kishk, tarhana, and super cereal plus corn–soy blend (SCpCSB) were compared for composition, color, water sorption, and reconstitution characteristics. FMBW blends had higher contents of protein, Ca, lactose and lactic acid, lower levels of salt (NaCl) and Fe, and a lighter, more‐yellow color (higher L* and b*‐color co‐ordinates) than tarhana or kishk. Compared with SCpCSB, FMBW had numerically higher levels of protein, lactose, and lactic acid, lower levels of Ca, Fe, Zn, and Mg, and lower pH. Tarhana had highest mean levels of starch, and on reconstitution (133 g/kg) had highest water holding capacity, viscosity during pasting and cooling, yield stress (*σ*_0_), consistency coefficient (*K*), and viscosity on shearing from 20 to 120 s^−1^ at 60°C. Reconstituted FMBW, kishk, and SCpCSB had similar pasting and flow behavior properties. Overall, the composition (starch, protein, Ca, Mg), pasting and flow behavior characteristics of FMBW were closer to those SCpCSB and kishk than to tarhana. The results suggest that the FMBW powder, on appropriate supplementation with Ca, Fe, Zn and Mg, could be used for the development of customized fortified blended foods for specific groups.

## 1 INTRODUCTION

Dehydrated milk‐cereal blends including super cereal plus (SCP), kishk or tarhana, are typically reconstituted and cooked to prepare soup and porridge, which are an important nutrient source for humans, especially for infants and young children (Black, Pahulu, & Dunn, 2009; Michaelsen et al., 2009; de Pee & Bloem, 2009). SCP is a category of fortified blended food (FBF) supplied by the World Food Programme (WFP) to improve the diet of young children, aged 6–59 months, in food insecure regions. It is prepared by dry‐blending heat‐treated cereal (wheat, corn, or rice), dehulled soybean, sugar, skim milk powder, refined soybean oil, vitamins, and minerals (WFP, 2015). The inclusion of milk solids in SCP enhances the levels of important nutrients such as essential amino acids, linoleic acid, fat soluble vitamins, and calcium (Michaelsen et al., 2009; O'Callaghan, Shevade, Guinee, O'Connor, & O'Brien, 2019). SCP complies to specifications for composition (e.g., protein, ≥160 g/kg; fat, ≥90 g/kg; calcium, 4,200–6,300 mg/kg), vitamins, physical characteristics (e.g., consistency—flow rate, 100 mm/30 s for 166.7 g/kg porridge), mycotoxins, and microorganisms (WFP, 2015).

Kishk is typically produced by blending yoghurt and parboiled wheat (bulgur) at a weight ratio of ~4:1, incubating the blend at ~35°C for 1–5 days, drying to ~70 g/kg moisture, and milling. Tarhana is produced using a similar approach to kishk but substitutes wheat bulgur with wheat flour, and additionally includes condiments such as seasonings, vegetables, and/or lentils (Cagindi, Aksoylu, Savlak, & Kose, 2016; Tamime, Barclay, Amarowicz, & McNulty, 1999). Both kishk and tarhana are consumed extensively in South Eastern Europe and Central Asia. The fermentation of the milk component, as in kishk and tarhana, further enhances the nutritional status of dairy‐cereal blends (Poutanen, Flander, & Katina, 2009; Rahmawati & Suntornsuk, 2016).

Surveys of commercial kishk (Salameh et al., 2016; Tamime et al., 1999) and tarhana (Cagindi et al., 2016; Simsek, Martinez, Daglioglu, Guner, & Gecgel, 2014) indicate significant inter‐ and intraproduct variation in composition, for example, starch and protein contents varied from ~420–590 and 140–220 g/kg, respectively, in kishk and ~600–700 and 30–150 g/kg, respectively, in tarhana. Despite the compositional variation, tarhana generally has a higher starch content and lower contents of protein, fat, lactic acid, salt, ash, and calcium than kishk. Such a trend suggests the use of a lower ratio of fermented milk‐to‐cereal in tarhana (Shevade, O'Callaghan, O'Brien, O'Connor, & Guinee, 2018). Analysis of data from Simsek et al. (2014) showed that the peak viscosity of reconstituted tarhana during pasting at 95°C and the breakdown viscosity on cooling to 50°C correlated positively with starch content. Similarly, Shevade et al. (2018) reported that starch content of reconstituted fermented milk‐wheat composites (65–87 g/kg) correlated positively with viscosity during pasting (95°C) and cooling (30°C), and with the flow behavior characteristics (yield stress; consistency index, and viscosity) on shearing from 20 to 120 s^−1^. Conversely, the latter parameters correlated negatively with contents of fat, protein, lactose, and lactic acid, all of which decreased as starch content increased (Shevade et al., 2018).

While various studies have analyzed the compositional and nutritional properties of commercial and experimental kishk (O'Callaghan et al., 2019; Salameh et al., 2016; Tamime et al., 1999), we are unaware of any studies on the pasting or rheological properties of reconstituted kishk, or of any comparative studies on the pasting or rheological properties of commercial tarhana and kishk. The nutritional value of liquid foods such as soup or porridge prepared from reconstituted milk‐cereal blends, including tarhana and kishk, is determined primarily by composition (Lee, Bello‐Pérez, Lin, Kim, & Hamaker, 2013; O'Callaghan et al., 2019) but is, nevertheless, likely to be influenced by texture and viscosity (Black et al., 2009; Fleige et al., 2010). An increase in viscosity delays gastric emptying, increases satiety and reduces hunger (Campbell, Wagoner, & Foegeding, 2017; Zhu, Hsu, & Hollis, 2013); moreover, viscosity of semiliquid foods affects their sensory appeal (Wendin et al., 2010). We are unaware of published information on the pasting behavior or rheology of reconstituted SCpCSB.

The objective of the current study was to develop an experimental dehydrated fortified blended food base product (FMBW) from a blend of fermented milk and wheat and with a gross composition and reconstitution behavior similar to SCpCSB, using the formulation and processing technology that has been traditionally applied in the manufacture of kishk and tarhana. To approach this objective, available commercial samples of SCpCSB, kishk and tarhana were analyzed for composition and consistency and the resultant data were used as a guide in formulation of the FMBW.

## 2 MATERIALS AND METHODS

### 2.1 Ingredients

Five commercial brands of tarhana powder and three of kishk powder were procured from Syria, Greece, Turkey, and Lebanon. Super cereal plus (corn–soy blend) was kindly supplied by Concern Worldwide.

Bulgur wheat (total solids, 906 g/kg; protein, 86 g/kg; fat, 45 g/kg; starch, 628 g/kg) was obtained from a local retail store (Tesco Sores Ltd.). Buttermilk powder (BMP; protein, 330 g/kg; fat, 70 g/kg; lactose, 460 g/kg; lactic acid, 2.3 g/kg) was obtained from Glanbia Ingredients Plc. Low heat skim milk powder (SMP; protein, 384.3 g/kg; fat, 8.9 g/kg; lactose, 462 g/kg) was manufactured using a pilot‐scale NIRO Tall‐Form Dryer in Moorepark Technology Limited (Teagasc), as described previously (Lin, Kelly, O'Mahony, & Guinee, 2016). Cream (fat, 351 g/kg; protein, 20 g/kg; TS, 408 g/kg) was purchased from a local retail store (Aldi Stores). Direct‐vat starter cultures CH1 YoFlex^®^ 207 (*Streptococcus thermophilus*) and YC380 (*Lactobacillus delbrueckii* subsp. *bulgaricus*) were obtained from Chr. Hansen Ireland Ltd.

### 2.2 Production of experimental fermented milk‐bulgur wheat blends (FMBW)

Fermented milk‐bulgur wheat blends were prepared, as described in detail by Shevade et al. (2018). Briefly, recombined milk (168 g/kg total solids) was formulated from a blend of reconstituted SMP (89 g/kg), BMP (70 g/kg), and cream (45 g/kg), heat‐treated at 95°C for 2.5 min, homogenized at first and second‐stage pressures of 15 and 5 MPa, respectively, cooled to 43°C (UHT/HTSTLab‐25 EHVH, MicroThermics^®^), inoculated with direct‐vat starter cultures, incubated at 42°C until the pH dropped to 4.6, cooled to 15°C, and stored at 4°C overnight. Fermented milk (FM) and bulgur wheat (1 mm particle size) were blended at a weight ratio of 80:20 for 5 min (Kenwood blender, Model KMM710 fitted K‐beater; Kenwood Ltd.), incubated at 35°C for 24 hr (Heratherm incubator, Thermo Fisher Scientific) to form a dough which was manually rolled into thin layers (0.5–1.0 cm) and dried for 48 hr at 46°C (Excalibur^®^ Dehydrator) to a moisture content of 60–70 g/kg. The resultant dried cake was manually broken into pieces and milled (Ultracentrifugal Mill ZM 200 fitted with a trapezoid 1 mm ring sieve; Retsch Technology GmbH) to a powder with a particle size of 1 mm, vacuum‐packed in polythene liners (Henkelman Polar 80 Floor Standing Vacuum Packer) and stored at 15°C.

The FMBW was produced on three separate occasions, using a separate batch of freshly prepared FM on each occasion.

### 2.3 Analysis of experimental and commercial dairy‐cereal powders

#### 2.3.1 Composition

Powders were analyzed in triplicate, as described by O'Callaghan et al. (2019) for protein by Kjeldahl, fat by Rose‐Gottlieb, NaCl by potentiometric determination of chlorine, moisture by drying to constant weight at 102°C, ash by heating at 550°C for 5 hr, calcium, iron, zinc, and magnesium by atomic absorption spectrophotometry (Varian, SpectrAA‐600). Starch, total dietary fiber, lactose, and lactic acid were determined using the following Megazyme K‐TSHK 09/15, KTDFR 12/15, K‐LACGAR, and K‐DLATE kits, respectively (Megazyme International Ireland). pH value was measured on a 5% aqueous dispersion of the powders prepared by stirring for 15 min at 21°C. Water Activity (*a*_w_) was measured at room temperature (Aqualab Dewpoint Water Activity Meter; Decagon Devices, Inc.).

#### 2.3.2 Color

The color space co‐ordinates, namely the L*, a*, and b* values, were measured on powdered samples using a CR‐400 Chroma Meter (Konica Minolta), which had been calibrated using the Minolta calibration plate. The L* value is indicative of lightness, varying from 0 (black) to 100 (white), whereas a*‐ and b*‐ values represent the variation in color from green (– values) to red (+ values), and of blue (– values) to yellow (+ values), respectively. A sample of powder (~20 g) was uniformly distributed in a Petri dish, and color measurements were made at three different locations along the sample surface.

#### 2.3.3 Water sorption

Water sorption and desorption behavior, as a function of relative humidity (RH) in the range 5%–85%, were measured gravimetrically using an SPS11 automatic multi‐sample moisture sorption analyzer (Project‐e Messtechnik, Enderlegasse), as described previously (Shevade et al., 2018). Powder samples (~500 mg) were subjected to a stepwise reduction in RH from 85% to 5% and thereafter to an increase in RH from 5% to 85%, at intervals of 10% RH. The results are expressed as water loss, or water uptake, per kg powder dry matter as a function of RH.

### 2.4 Analysis of reconstituted experimental and commercial dairy‐cereal powders

#### 2.4.1 Water holding capacity (WHC)

Powders (133 g/kg) were reconstituted in distilled water, cooked (heated to 95°C over 10 min, held at 95°C for 25 min), cooled to 21°C while stirring at 120 rpm, and centrifuged (Sorvall Lynx 6000 Superspeed centrifuge [ThermoElectron LED GmbH]) at 12,500 *g* for 1 hr at 20°C (Shevade et al., 2018). WHC was defined as the weight of the pellet, expressed as g/kg of reconstituted powder before centrifugation.

#### 2.4.2 Gelatinization temperature

Gelatinization characteristics were analyzed in triplicate using differential scanning calorimetry (DSC 2000, TA instruments) as described by Shevade et al. (2018). Triplicate samples of each powder were reconstituted in distilled water in a quantity sufficient to obtain a water‐to‐starch ratio of 11.4:1.0, stirred for 15 min (IKA^®^ RT 10 Magnetic Stirrer, IKA‐Werke GmbH) at room temperature, loaded in the calorimeter, and scanned on heating from 20 to 95°C at 5°C/min. An empty pan was used as a reference. The temperatures at gelatinization onset (*T*_o_), peak (*T*_p_), and end (*T*_e_) were obtained for each sample endotherm from the system software (TA Universal Analysis).

#### 2.4.3 Pasting behavior

Reconstituted powder (133 g/kg) was pasted in a controlled stress rheometer (Anton Paar Physica MCR 501 Rheometer, Anton Paar GmbH), fitted with a starch pasting cell, comprising a measuring cup and stirrer, as described previously (Shevade et al., 2018). The sample was tempered at 25°C for 1 min, sheared at 160 s^−1^, heated to 95°C over 10 min, held at 95°C for 25 min, and cooled to 30°C in 10 min while constantly shearing (160 s^−1^). The viscosity was measured dynamically and the following parameters were calculated from the resultant viscosity/time curves: peak viscosity (*V*_p_); viscosity after heating to 95°C (*V*_95_), holding at 95°C (*V*_h_) and cooling to 30°C (*V*_c_); breakdown viscosity (BRV = *V*_p_ − *V*_h_) which denotes the viscosity decrease during holding; and setback viscosity (SBV = *V*_c_ − *V*_h_) corresponding to the viscosity increase during cooling.

#### 2.4.4 Flowability

Reconstituted powder (133 g/kg) was cooked, as described for WHC, and cooled to 46°C in 5 min while constantly stirring (120 rpm). A sample of the cooled soup (120 g) was loaded into the chamber of a Bostwick Consistometer (CR Instruments Limited), which was preheated to 46°C. The extent flow (mm) in 30 s on release of the sample from the chamber was recorded (Shevade et al., 2019).

#### 2.4.5 Rheology

Powder was reconstituted (133 g/kg), cooked, and cooled to 60°C while stirring at 120 rpm. Rheology was measured in a subsample (11 g) of the cooled product as described previously (Shevade et al., 2018). The sample was placed in the measuring cell of a rheometer (Carri‐Med, type CSL2500, TA instruments), consisting of an outer cup (diameter 27.5 mm) and an inner bob (diameter 25 mm). Following equilibration at 60°C for 5 min, the sample was subjected to shear rate ($\dot{\gamma }$) sweep, from 18 to 120 s^−1^ over 20 min. The changes in shear stress (***σ***; Pa) and viscosity (***η***; Pa.s) were recorded. The resultant σ versus $\dot{\gamma }$ data were fitted to the Herschel–Bulkley model using TA data analysis software (TA Rheology Advance Data Analysis, Version V5.7.0):

(1) $$\sigma =\sigma_{0}+K{\dot{\gamma }}^{n}$$

where *σ*_0_, *K*, and *n* represent yield stress (Pa), consistency coefficient (Pa.s), and flow behavior index (*n*), respectively (Aguilar‐Raymundo & Vélez‐Ruiz, 2018).

### 2.5 Statistical analysis

The data were analyzed using a randomized complete block design which incorporated the three different powder types and replicate samples of each powder type (3 for FMBW, 5 for tarhana, 3 for kishk). Analysis of variance (ANOVA) was carried out using the general linear model (GLM) procedure of SAS 9.3 to determine the effect of powder type on each response variable (SAS, 2011). Tukey's multiple‐comparison test was used for paired comparison of treatment means and the level of significance was determined at *p* < .05. Data for Super cereal *plus* corn–soy blend (SCpCSB) were included as an observation.

## 3 RESULTS AND DISCUSSION

### 3.1 Properties of experimental and commercially available dairy‐cereal powders

#### 3.1.1 Gross composition

The levels of fat, protein, and starch in the kishk and tarhana powders were within the range reported previously for experimental tarhana (Bilgiçli, 2009) and commercial kishk (Salameh et al., 2016; Tamime et al., 1999) powders. However, gross composition differed significantly with powder type (Table 1), with interpowder variation being relatively high for lactose, lactic acid, salt, and calcium (coefficient of variation, cv = ≥ ~70%), intermediate for fat, protein, starch, moisture, ash, Zn and Mg (cv = ~20%–40%), and low for dry matter, and pH (cv < 9%). Such variation is indicative of differences in formulation, which provide a means of controlling cost and customization of the powders to different end‐use applications and markets.

**Table 1:** Composition of dairy‐cereal powders: experimental fermented milk‐bulgur wheat blend (FMBW) and commercial samples of tarhana, kishk, and super cereal plus corn–soy blend (SCpCSB)

Item	FMBW	Tarhana	Kishk	SCpCSB
Powder composition				
Dry matter (g/kg)	930^a^ ± 6	891^b^ ± 27	907^ab^ ± 3	954
Protein (g/kg)	196^a^ ± 5	100^c^ ± 13	156^b^ ± 39	164
Fat (g/kg)	59^ab^ ± 2	40^b^ ± 10	91^a^ ± 62	104
Starch (g/kg)	384^b^ ± 11	624^a^ ± 35	467^b^ ± 101	372
Lactose (g/kg)	70^a^ ± 1	9^b^ ± 5.2	13^b^ ± 18	34
Ash (g/kg)	43^b^ ± 6.0	29^c^ ± 15	55^a^ ± 11	39
Salt (g/kg)	7.6^c^ ± 0.4	21.8^b^ ± 16.5	42.1^a^ ± 13.8	3.3
Lactic acid (g/kg)	48.8^a^ ± 1.8	8.6^c^ ± 5.2	22.9^b^ ± 4.2	0.1
Calcium (mg/kg)	2,968^a^ ± 148	328^c^ ± 139	1,712^b^ ± 608	4,628
Iron (mg/kg)	8.9^c^ ± 0.1	29.0^b^ ± 6.8	49.6^a^ ± 23.0	128.0
Zinc (mg/kg)	20^b^ ± 0	12^c^ ± 2	28^a^ ± 5	82
Magnesium (mg/kg)	668^b^ ± 14	451^c^ ± 76	919^a^ ± 145	1,117
pH	3.9^b^ ± 0.0	4.6^a^ ± 0.8	4.0^b^ ± 0.2	6.7
Color space co‐ordinates†				
L*	90.5^a^ ± 0.5	63.5^b^ ± 4.0	67.4^b^ ± 4.3	80.5
a*	−1.2^b^ ± 0.1	5.4^a^ ± 5.6	0.01^ab^ ± 0.9	−0.7
b*	25.8^a^ ± 0.6	23.7^ab^ ± 7.7	15.8^b^ ± 4.9	37.2
Water sorption† (water g/kg Dry matter)				
85% RH	314^a^ ± 7	272^a^ ± 54	311^a^ ± 37	216
5% RH	40^a^ ± 1	50.5^a^ ± 16	37^a^ ± 5	26
Water activity, *a*_w_	0.42^a^ ± 0.03	0.58^a^ ± 0.14	0.53^a^ ± 0.03	0.41

Note

^a–c^Values within a row relating to FMBW, tarhana, and kishk and not sharing a common lower‐case superscripted letter differ significantly (*p* < .05). Presented data are mean values ± standard deviations for 3 replicate trials of FMBW, 5 for tarhana, and 3 for kishk; data for 1 sample of commercial SCpCSB are included as observations.

Abbreviation: RH, relative humidity.

^†^ L*, lightness; a*, green to red; and b*, blue to yellow.

Fermented milk‐bulgur wheat powder (FMBW) had significantly higher mean levels of protein, lactose, lactic acid and Ca, and lower mean contents of salt and Fe, than tarhana or kishk powders. Compared with tarhana, kishk had higher mean levels of protein, fat, lactic acid, salt, ash, Ca, Fe, and Zn, and a lower mean content of starch. Overall, tarhana had lowest mean levels of protein, fat, lactic acid, Ca, Mg, Zn, ash, highest mean starch content, and pH. The data suggest a higher ratio of fermented milk‐to‐wheat in FMBW than in kishk or tarhana (Shevade et al., 2018). This is supported by the absence of lactose in wheat, and the higher concentrations of protein and Ca, on a dry matter basis, in milk than in wheat (Gulati et al., 2018; King, Zeug, & Pettit, 2010). Moreover, the inclusion of vegetables, which have a relatively low protein‐in‐dry matter content, as an ingredient in commercial tarhana (O'Callaghan et al., 2019) is likely to further reduce the contents of protein and fat.

Super cereal plus corn–soy blend had numerically higher mean values of fat, Ca, Mg, Fe, Zn, and pH, and lower lactic acid than the other powders. This observation is consistent with the addition of refined soybean oil, vitamin/mineral mixture, and nonfat dried milk solids to SCpCSB to enhance levels of essential fatty acids (linoleic and linolenic acids) and minerals (WFP, 2015).

#### 3.1.2 Color of powders

The color co‐ordinates (L*, a*, b*) of the powders are shown in Table 1. The values of L* (63.5–90.5), a* (−0.7 to 5.4) and b* (15.8–37.2) for all powders were comparable to those previously reported for kishk and tarhana, i.e., 48–89, −0.14 to 28.1, and 1.43–52.88, respectively (Cagindi et al., 2016; Salameh et al., 2016).

Fermented milk‐bulgur wheat had significantly higher mean values of L* and b* than kishk, and higher and lower values L* and a*, respectively, than tarhana. The difference in color co‐ordinates correlated with the visual appearance which indicated that the FMBW was more yellow than either kishk or tarhana, and less red than tarhana. Color differences most likely reflect differences in formulation, for example, types and proportions of ingredients, and processing conditions, for example, time and temperature during parboiling the wheat kernels (Mir & Bosco, 2013; de Pee & Bloem, 2009). The inclusion of red pepper and tomato, which contain high levels of the carotenoids β‐carotene, capsanthin, lutein, and zeaxanthin (O'Callaghan et al., 2019), in three of the tarhana samples, is likely to have contributed to its relatively high mean a* value, compared to kishk and FMBW. Overall, FMBW was closer in appearance to SCpCSB, being relatively light and yellow compared to kishk and tarhana.

#### 3.1.3 Water sorption characteristics

**Figure 1:**
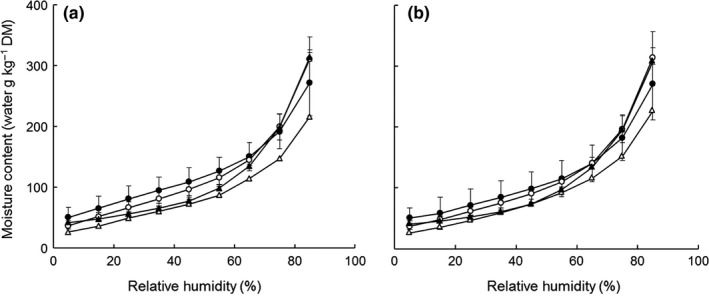
Sorption isotherms for dairy‐cereal powders: experimental fermented milk‐bulgur wheat blend, FMBW (▲), and commercial samples of tarhana (●), kishk (○) and super cereal plus corn–soy blend, SCpCSB (∆), during (a) desorption and (b) adsorption. Presented values are the means of 3 replicate samples of FMBW, 5 for tarhana, and 3 for kishk; error bars represent *SD* of the mean. Data for 1 sample of SCpCSB are included as an observation

The water desorption and adsorption isotherms of the powders at 5%–85% RH and 20°C are shown in Figure 1. On initial equilibrium at 85% RH, the moisture‐in‐dry matter (MDM) content of all powders increased from initial values of ~50–110 to 216.1–314.4 g/kg depending on the powder (Table 1). The water uptake at 85% RH indicates the adsorption of water molecules to the surface of powder particles, for example, by capillary condensation or interaction with hydrophilic (charged and polar) groups of surface constituents through hydrogen bonding (Al‐Muhtaseb, McMinn, & Magee, 2002). The MDM content decreased to ~25–50 g/kg on lowering RH to 5%, with the decrease being most pronounced in the 85%–55% RH zone, and more gradual thereafter (Figure 1a). On increasing the RH again to 85%, the MDM reverted to original values with equilibrium values at each RH coinciding with those on desorption (Figure 1b). No hysteresis was observed between desorption and adsorption curves with respect to RH, suggesting that any changes in particle structure during desorption (e.g., as a result of protein–protein or protein–mineral interactions, salt deposition) were fully reversible and did not affect water sorption.

Despite the differences in composition (Table 1), the mean equilibrium MDM content of the FMBW, tarhana, and kishk powders at 85% RH and 5% RH did not significantly differ. Shevade et al. (2018) found that the equilibrium MDM of experimental fermented milk‐wheat powder blends at 85% RH and 5% RH increased significantly with incremental increase in sugar content (lactose and galactose) from ~85 to 150 g/kg and simultaneous reduction in starch content from 520 to 390 g/kg as the ratio of fermented milk‐to‐cereal in the blend was increased. The authors attributed this effect to the increase in concentration of low molecular weight of sugars, which was conducive to a higher number of hydroxyl groups and, hence, hydrogen bonding with water, per unit weight of sugar than starch. However, while a similar trend was observed in the current study, the differences between FMBW, tarhana, and kishk powders were nonsignificant.

The equilibrium MDM values for SCpCSB at all RH values were notably lower than those of the other powders. This may reflect differences in composition, type of cereal used in formulation (e.g., wheat vs. maize), and characteristics of starch granules (presence of amylose‐lipid complexes, size/shape), porosity, and processing conditions (e.g., parboiling, shearing; Al‐Muhtaseb et al., 2002; Al‐Muhtaseb, McMinn, & Magee, 2004). A contributory factor for the relatively low equilibrium MDM values of SCpCSB may be its low contents of starch, lactose, and lactic acid (Table 1); the latter compounds have a relatively high proportion of hydroxyl groups which have a high affinity to hydrogen bond with water molecules.

### 3.2 Properties of reconstituted experimental and commercially available dairy‐cereal powders

#### 3.2.1 Gelatinization temperature

Gelatinization of native starch is an endothermic process, whereby heating results in the heat‐induced disruption of hydrogen bonds (otherwise maintaining the crystalline regions of the starch granule intact), hydration of starch molecules, granule swelling, and an increase in viscosity (Alcázar‐Alay & Meireles, 2015; Wani et al., 2012). It is accompanied by an endothermic phase transition peak typically between ~52 and 76°C (Singh, Singh, Kaur, Sodhi, & Gill, 2003), with the range of gelatinization temperature depending on starch type, which affects the granule morphology and molecular structure, for example, degree of crystallinity and level of phospholipids.

None of the reconstituted powders, apart from tarhana (Table 2), underwent gelatinization, as indicated by the absence of an endothermic phase transition peak on heating from 20 to 95°C (Figure S1a,b). A similar trend was observed by Shevade et al. (2018) and Shevade et al. (2019) for fermented milk‐cereal composites, prepared using different cereal types and with varying ratio of FM to cereal. The results suggest that the starch in kishk and SCpCSB may have been gelatinized by heating/parboiling of the cereal component prior to formulation (Tamime, Muir, Khaskheli, & Barclay, 2000). Sittipod and Shi (2016) found that preheating of rice flour at high temperature (i.e., 110–120°C) for 60–70 min resulted in the loss of the heat endotherm during subsequent heating. The occurrence of a heat endotherm between 57 and 75°C for tarhana suggests that the wheat flour used in formulation did not undergo a preheat treatment (O'Callaghan et al., 2019).

**Table 2:** Reconstitution characteristics of dairy‐cereal powders: experimental fermented milk‐bulgur wheat blend (FMBW) and commercial samples of tarhana, kishk, and super cereal plus corn–soy blend (SCpCSB)

Item	FMBW	Tarhana	Kishk	SCpCSB
Gelatinization†				
*T*_o_ (°C)	–	58 ± 4	–	–
*T*_p_ (°C)	–	66 ± 3	–	–
*T*_e_ (°C)	–	75 ± 2	–	–
Water holding capacity (pellet, g/kg)				
45 min	509^b^ ± 10	999^a^ ± 2	565^b^ ± 162	602
Pasting/cooling viscosity†				
*V*_95_ (Pas)	0.04^b^ ± 0.02	2.90^a^ ± 0.99	0.17^b^ ± 0.11	0.11
*V*_p_ (Pas)	–	2.99^a^ ± 0.87	–	0.13
*V*_h_ (Pas)	0.08^b^ ± 0.03	1.37^a^ ± 0.37	0.22^b^ ± 0.21	0.09
*V*_c_ (Pas)	0.26^b^ ± 0.08	2.95^a^ ± 1.37	0.61^b^ ± 0.53	0.34
BRV (Pas)	–	1.61 ± 0.72	–	0.04
SBV (Pas)	0.18^b^ ± 0.05	1.58^a^ ± 1.44	0.39^ab^ ± 0.32	0.25
Pasting time (min)	–	10.98 ± 0.16	–	9.60
Rheology†				
*σ*_0_ (Pa)	2.27^b^ ± 1.11	61.2^a^ ± 14.00	6.49^b^ ± 6.98	1.52
*K* (Pa.s^n^)	0.79^b^ ± 0.90	26.75^a^ ± 7.56	2.96^b^ ± 2.59	0.21
*n* (−)	0.64^a^ ± 0.05	0.68^a^ ± 0.34	0.63^a^ ± 0.33	0.80
*η* at 120 s^−1^ (Pas)	0.07^b^ ± 0.06	1.15^a^ ± 0.59	0.21^b^ ± 0.17	0.08
Flowability (mm/30sec)	231.3^a^ ± 0.3	48.0^b^ ± 0.5	183.9^a^ ± 5.8	235.0

Note

^a–c^Values within a row relating to FMBW, tarhana, and kishk and not sharing a common lower‐case superscripted letter differ significantly (*p* < .05); blank (−) indicates the absence of values for specified characteristics. Presented data are mean values ± standard deviations for 3 replicate samples of FMBW, 5 for tarhana, and 3 for kishk; data for 1 sample of commercial SCpCSB are included as observations.

^†^ *T*_o_, *T*_p_, and *T*_e_ correspond to temperatures at the onset, peak, and end of gelatinization, respectively; *V*_95_, *V*_h_, and *V*_c_ denote viscosity after heating at 95°C over 10 min, holding at 95°C for 25 min, and cooling to 30°C over 10 min, respectively; *V*_P_, BRV, and SBV refer to peak viscosity during heating and holding, viscosity decrease on holding at 95°C (*V*_p_ − *V*_h_), and viscosity increasing during cooling (*V*_c_ − *V*_h_), respectively; *σ*_0_, *K*, *n*, and $\eta_{120\;s^{-1}}$ correspond to yield stress, consistency index, flow behavior index, and viscosity at 120 s^−1^, respectively, on shearing from 20 to 120 s^−1^.

#### 3.2.2 Water holding capacity (WHC)

Water holding capacity is indicative of an increase in water absorption and swelling of the reconstituted powder (particles) during stirring and heating, and hence is likely to impact on the viscosity of the resultant soup/porridge (Wani et al., 2012).

Significant differences were observed for WHC after cooking, with the mean value for tarhana being significantly higher than those for kishk and FMBW, and numerically higher than that of SCpCSB (Table 2). The higher WHC of tarhana most likely reflects its higher starch content (Table 1), which correlated positively with WHC (Table 3; *R* = .91, *n* = 28), and its relatively low concentration of lactic acid which correlated negatively with WHC. The results are consistent with those of other studies which reported an increase in the storage modulus, G′, of wheat, maize, and potato starch pastes as starch content was increased in the range 75–125 g/kg (Eliasson, 1986), which is inclusive of the spread in levels (49–83 g/kg) in the reconstituted products in the current study. Sriburi and Hill (2000) found that the viscosity (90°C) of cassava starch extrudates, prepared with different levels of added ascorbic acid, decreased progressively with pH reduction from 7.0 to 3.5, and suggested acid‐induced starch hydrolysis as the causative factor.

**Table 3:** Correlations between powder composition and characteristics of the reconstituted powder

Characteristic	Composition (g/kg)					
	Starch	Protein	Fat	Lactose	Lactic acid	Salt
Water holding capacity (pellet, g/kg)						
At 45 min	0.91***	−0.94***	−0.77***	−0.51	−0.57***	−0.11
Pasting/cooling viscosity†						
*V*_95_ (Pas)	0.84***	−0.76***	−0.51**	−0.43*	−0.51**	−0.26
*V*_p_ (Pas)	0.84***	−0.77***	−0.52**	−0.43*	−0.53**	−0.26
*V*_h_ (Pas)	0.85***	−0.83***	−0.61***	−0.52*	−0.49*	−0.04
*V*_c_ (Pas)	0.77***	−0.64***	−0.49**	−0.19	−0.56*	−0.26
BRV (Pas)	0.75***	−0.64**	−0.40*	−0.35	−0.50**	−0.10
SBV (Pas)	0.53*	−0.37	−0.29	−0.16	−0.45**	−0.34
Rheology†						
*σ*_0_ (Pa)	0.84***	−0.76***	−0.61***	−0.43	−0.58**	−0.02
*K* (Pa.s^n^)	0.83***	−0.79***	−0.56*	−0.37	−0.74***	−0.18
*n* (−)	0.51*	−0.62**	−0.62***	−0.18	−0.34	−0.02
*η* at 120 s^−1^ (Pas)	0.79***	−0.62**	−0.47**	−0.35	−0.53*	0.03
Flowability (mm/30 s)	−0.87***	0.93***	0.72***	0.39*	0.66***	−0.09

Note

Correlations were obtained using simple linear regression analysis of the entire data set, relating to 3 samples of experimental fermented milk‐bulgur wheat blend (FMBW), 5 of tarhana, 3 of kishk, and 1 of super cereal plus corn–soy blend (SCpCSB). Negative correlations between two parameters are indicated by a negative sign (−).

^†^ *V*_95_, *V*_h_, and *V*_c_ denote viscosity after heating at 95°C over 10 min, holding at 95°C for 25 min, and cooling to 30°C over 10 min, respectively; *V*_p_, BRV, SBV refer to peak viscosity during heating and holding, viscosity decrease on holding at 95°C (*V*_p_ − *V*_h_), and viscosity increasing during cooling (*V*_c_ − *V*_h_), respectively; *σ*_0_, *K*, *n*, and $\eta_{120\;s^{-1}}$ correspond to yield stress, consistency index, flow behavior index, and viscosity at 120 s^−1^ respectively, on shearing from 20 to 120 s^−1^.

*** *p* < .001; **p < .01; *p < .05: Significance levels.

However, the WHC is also likely to be influenced by compositional factors, other than starch content, such as starch granule dimensions, relative proportions of amylose‐to‐amylopectin, and the presence of other components including proteins, lipids, salt, and acids, and their interactive effects (Kett et al., 2013; Kumar, Brennan, Zheng, & Brennan, 2018; Singh et al., 2003).

#### 3.2.3 Pasting behavior

**Figure 2:**
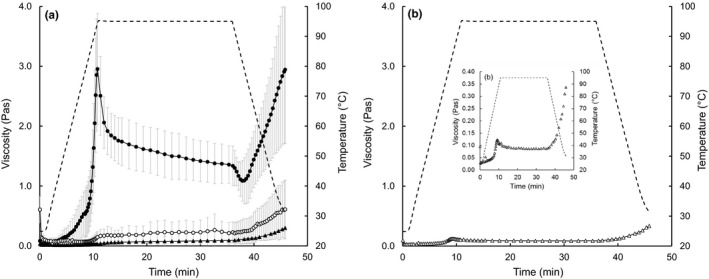
Changes in viscosity (solid lines) and temperature (broken line) during pasting (cooking) and cooling of reconstituted dairy‐cereal powders: (a) experimental fermented milk‐bulgur wheat blend, FMBW (▲), commercial samples of tarhana (●) and kishk (○), and (b) super cereal plus corn–soy blend, SCpCSB (∆); the inset in (b) shows changes during pasting of SCpCSB in more detail. Presented values are the means of 3 replicate samples of FMBW, 5 for tarhana, and 3 for kishk; error bars represent *SD* of the mean. Data for 1 sample of SCpCSB are included as an observation

The viscosity of the reconstituted powders during pasting (heating and holding at 95°C) and cooling to 30°C is shown Figure 2. The viscosity–time profile of reconstituted tarhana was similar to that of native starch dispersions (Wani et al., 2012), which features a peak‐, breakdown‐, and setback‐ viscosity (Figure 2a). The viscosity increased steeply to *V*_p_ on heating, decreased to *V*_h_ on holding at 95°C, and increased to *V*_c_ on cooling to 30°C. The SCpCSB showed a similar pattern, though the viscosity was significantly lower at all stages of pasting and cooling (Figure 2b). Factors contributing to viscosity changes include disruption of hydrogen bonding between starch molecules followed by hydration and swelling of the starch granules (during heating), partial granule fracture coinciding with dissociation of starch molecules (especially amylose from the amorphous regions of the granule) to the surrounding aqueous phase on holding, and re‐association of amylose molecules into aggregates during cooling (Wang, Li, Copeland, Niu, & Wang, 2015; Wani et al., 2012). In contrast to tarhana, the viscosity of the reconstituted FMBW and kishk remained relatively constant during heating, and increased progressively, but slowly, during holding and cooling (Figure 2a). The difference in viscosity profile/time between tarhana or SCpCSB and FMBW or kishk suggests a pregelatinization effect of parboiling the wheat used in the formulation of FMBW and kishk, for example, 95°C for 60–70 min (Majzoobi & Beparva, 2014; Shevade et al., 2018). This is supported by the absence of a DSC endothermic peak on heating FMBW or kishk to 95°C (Figure S1a,b). Parboiling typically involves heating the cereal kernels to temperatures of 30–100°C by soaking in hot water for 0.5–24 hr, or subjecting to steaming for 4–30 min (Tamime et al., 2000) and subsequently drying. It is conducive to pregelatinization and granule rupture to an extent dependent on temperature and duration (Dutta, Mahanta, & Singh, 2015; Mir & Bosco, 2013). Increasing the temperature of parboiling from 30 to 95°C has been found to reduce the viscosity of the reconstituted parboiled cereal at all stages of pasting and cooling, and leads to the eventual disappearance of a peak viscosity from the viscosity/time profile (Mir & Bosco, 2013). The presence of *V*_p_ and BRV in the viscosity–time profile of SCpCSB, even though the formulation specifies the use of heat‐treated cereal (WFP, 2015), suggests a relatively low heat treatment of the cereal kernels, and consequently, a low degree of starch granule rupture; it is possible that the heat treatment corresponds to a dry‐heat parboiling with relatively low heat treatment (e.g., <140°C for <10 min; Dutta et al., 2015).

Tarhana had higher mean values of *V*_95_, *V*_h_, *V*_c_, BRV, and SBV than FMBW, kishk, or SCpCSB (Table 2). Regression analysis indicated that the latter parameters correlated positively with starch content and negatively with lactic acid content (Table 3). The higher *V*_95_ and *V*_h_ are indicative of a higher degree of granule swelling (Kumar et al., 2018; Wani et al., 2012) and are consistent with its higher WHC at 45 min (Table 2). Apart from starch content and extent of parboiling treatment, differences in composition (e.g., concentrations of protein, fat, lactose, and lactic acid), which affect factors such as available water, the degree of granule fracture, proportion of soluble amylose, and degree of starch hydrolysis prior to cooling (Alcázar‐Alay & Meireles, 2015; Ghasemi, Mosavian, & Khodaparast, 2008; Kumar et al., 2018), are also likely to impact viscosity on heating and cooling. Tarhana had relatively high and low contents of starch and lactic acid, respectively, and it is likely that these may in part have contributed to its higher viscosity values (Ghasemi et al., 2008; Majzoobi & Beparva, 2014; Simsek et al., 2014). The positive effect of starch content is consistent with the results of Shevade et al. (2018) who reported positive correlations between the starch content (60–90 g/kg) and *V*_p_, *V*_h_, *V*_c_, BRV, and SBV of native wheat starch dispersions. Acidification of starch dispersions prior to heating has been found to increase starch hydrolysis and reduce granule integrity, pasting temperature, and viscosity to an extent depending on the degree of acidification (Ghasemi et al., 2008; Ohishi, Kasai, Shimada, & Hatae, 2007). Majzoobi and Beparva (2014) reported that the addition of concentrated lactic acid (150 mg/kg starch) to native‐ or pregelatinized‐starch dispersions (400 g/kg) resulted in significant reductions in intrinsic viscosity, equivalent to ~29% for the former and 43% for the latter; the effect was attributed to starch granule deformation.

Setback during cooling is indicative of retrogradation, in response to the intermolecular re‐association of solubilized starch molecules, especially amylose, through hydrogen bonding (Wang et al., 2015). The relatively high SBV of tarhana concurs with the findings of Eliasson (1986) and may reflect its higher starch content, and, hence its higher expected amylose level. However, as for *V*_95_ and *V*_h_, retrogradation of starch‐based suspensions is also affected by compositional factors other than starch content (Wang et al., 2015).

The relatively high values of *V*_95_, *V*_h_, and *V*_c_ of the reconstituted tarhana was confirmed by tactile and visual observation which showed it to be thicker during cooking and more stodgy after cooling than the other reconstituted powders. Overall, the consistency of cooled tarhana was porridge‐like, while that of the cooled FMBW, kishk and SCpCSB were soup‐like. Hence, the flowability of the cooked tarhana immediately after cooling to 46°C, as measured using the Bostwick consistometer, was notably lower than that of the other powders (Table 2).

#### 3.2.4 Rheology

**Figure 3:**
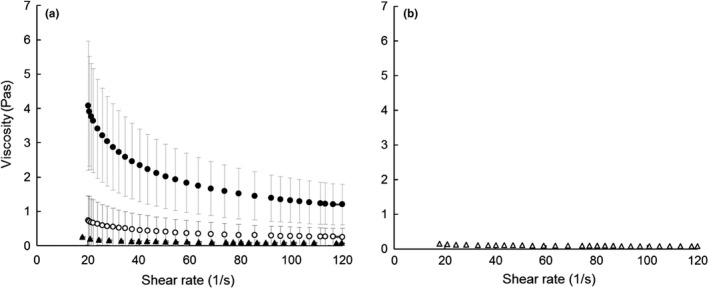
Flow curves at 60°C for reconstituted dairy‐cereal powders: (a) experimental fermented milk‐bulgur wheat blend, FMBW (▲), commercial samples tarhana (●), kishk (○), and (b) super cereal plus corn–soy blend, SCpCSB (∆). Presented values are the means of 3 replicate samples of FMBW, 5 for tarhana, and 3 for kishk; error bars represent *SD* of the mean. Data for 1 sample of SCpCSB are included as an observation

The reconstituted powders were cooked at 95°C, cooled to 60°C, and subjected to shear rate sweep from 18 to 120 s^−1^. The shear rate versus shear stress data for each powder fitted to the Herschel–Bulkley model (*R* > .99). All products displayed a yield stress (*σ*_0_) and shear thinned (Figure 3), suggesting shear‐induced degradation of an internal network, most likely comprised of starch and protein (Shevade et al., 2019).

The reconstituted tarhana had significantly higher mean values of yield stress (*σ*_0_), *K* and $\eta_{120\;s^{-1}}$ than FMBW or kishk, which were similar (Figure 3a). Otherwise, all powders had similar values of *n*, indicating a similar rate of shear thinning for all samples. The higher viscosity of tarhana over the entire shear rate is consistent with its higher WHC and pasting viscosity, and lower flowability. Linear regression analysis of the entire data set showed that most of the rheological parameters correlated positively with starch content and negatively with levels of fat, protein, and lactic acid (Table 3); this trend suggests that substitution of starch with the latter components leads to a lower‐viscosity soup or porridge. The impact of starch concentration on rheology is consistent with the findings of Aguilar‐Raymundo and Vélez‐Ruiz (2018) who found that an incremental increase in the concentration of chickpea flour (83–113 g/kg) in dairy desert coincided with increases in *σ*_0_ and *K*.

While SCpCSB had numerically lower values of *σ*_0_ and *K* and $\eta_{120\;s^{-1}}$ than the other samples, it resembled FMBW most closely (Figure 3b).

## 4 CONCLUSIONS

An experimental fortified blended food base product (FMBW) was prepared by blending fermented milk (168 g/kg total solids) with parboiled dehulled wheat (906 g/kg total solids) at a weight ratio of 4:1 and drying the blend (930 g/kg total solids). The composition and reconstitution behavior of the FMBW were compared with those of commercial dehydrated dairy‐cereal blends, namely super cereal plus corn–soy blend (SCpCSB), tarhana and kishk. Compared with SCpCSB, FMBW had numerically higher contents of protein, lactose, lactic acid, and salt, lower levels of fat, Ca, Mg, Fe, and Zn, and similar pasting viscosity profile, and flow behavior properties (yield stress, *σ*_0_; consistency coefficient, *K*; and viscosity) when reconstituted (133 g/kg) and cooked as a soup. Tarhana powders had highest mean values of starch and pH, lowest contents of lactic acid and Ca, and highest values of water holding capacity (WHC) and viscosity on reconstitution and cooking. The kishk samples were closer to FMBW and SCpCSB than to tarhana for most of the measured parameters. The results highlight the importance of formulation on the composition of dehydrated fermented milk‐cereal blends and their consistency when reconstituted. Consistency is likely to impact on consumer acceptability and satiety aspects of the reconstituted powders when cooked and, hence, their effectiveness as a nutrient vector.

## CONFLICT OF INTEREST

The authors have no conflict of interest.

## ETHICAL APPROVAL

This study did not involve any human or animal testing.
