# Supplementary figures and images for: Development of a dehydrated fortified food base from fermented milk and parboiled wheat, and comparison of its composition and reconstitution behavior with those of commercial dried dairy‐cereal blends
Source: Food Sci Nutr. 2019 Oct 15;7(11):3681–91. doi: 10.1002/fsn3.1226 (PMC6848806; doi:10.1002/fsn3.1226)

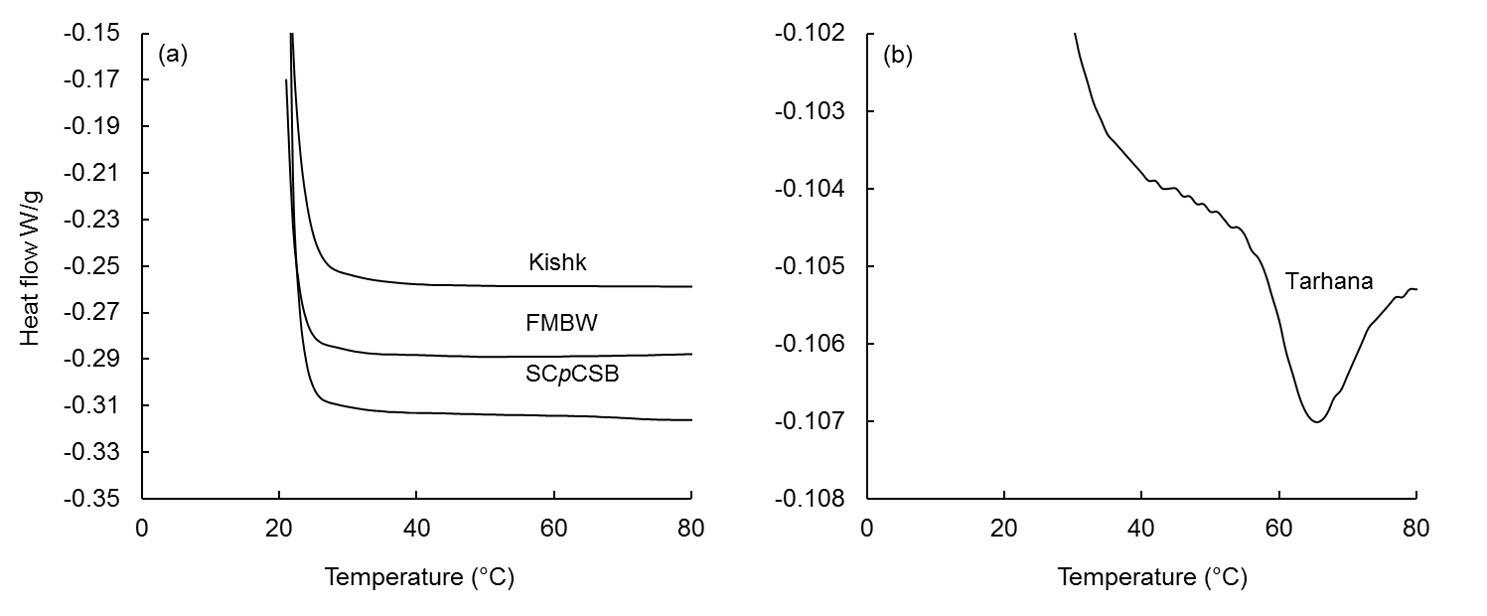

Supplement: Supplementary file 1 [file FSN3-7-3681-s001.tif]
