# Supplementary material for: Development of a dehydrated fortified food base from fermented milk and parboiled wheat, and comparison of its composition and reconstitution behavior with those of commercial dried dairy‐cereal blends
Source: Food Sci Nutr. 2019 Oct 15;7(11):3681–91. doi: 10.1002/fsn3.1226 (PMC6848806; doi:10.1002/fsn3.1226)
Supplement: Supplementary file 2 [file FSN3-7-3681-s002.docx]

Figure S1. Differential scanning calorimetry endotherms (DSC) for dairy-cereal powders: (a) experimental fermented milk-bulgur wheat blend (FMBW) and commercial samples of kishk and super cereal plus corn soya blend ( SCpCSB), and (b) tarhana.
